# Transcriptomic signature of Bexarotene (Rexinoid LGD1069) on mammary gland from three transgenic mouse mammary cancer models

**DOI:** 10.1186/1755-8794-1-40

**Published:** 2008-09-11

**Authors:** Martin C Abba, Yuhui Hu, Carla C Levy, Sally Gaddis, Frances S Kittrell, Yun Zhang, Jamal Hill, Reid P Bissonnette, Daniel Medina, Powel H Brown, C Marcelo Aldaz

**Affiliations:** 1 Department of Carcinogenesis, The University of Texas M.D. Anderson Cancer Center, Science Park-Research Division, Smithville, 78957, TX, USA; 2 Breast Center, Departments of Medicine and Molecular and Cellular Biology, Baylor College of Medicine, Houston, 77030, TX, USA; 3 Department of Molecular Oncology, Ligand Pharmaceuticals Inc., San Diego, California, 92121, USA; 4 CINIBA, Facultad de Ciencias Médicas, Universidad Nacional de La Plata, La Plata, Argentina

## Abstract

**Background:**

The rexinoid bexarotene (LGD1069, Targretin) is a highly selective retinoid × receptor (RXR) agonist that inhibits the growth of pre-malignant and malignant breast cells. Bexarotene was shown to suppress the development of breast cancer in transgenic mice models without side effects. The chemopreventive effects of bexarotene are due to transcriptional modulation of cell proliferation, differentiation and apoptosis. Our goal in the present study was to obtain a profile of the genes modulated by bexarotene on mammary gland from three transgenic mouse mammary cancer models in an effort to elucidate its molecular mechanism of action and for the identification of biomarkers of effectiveness.

**Methods:**

Serial analysis of gene expression (SAGE) was employed to profile the transcriptome of p53-null, MMTV-ErbB2, and C3(1)-SV40 mammary cells obtained from mice treated with bexarotene and their corresponding controls.

**Results:**

This resulted in a dataset of approximately 360,000 transcript tags representing over 20,000 mRNAs from a total of 6 different SAGE libraries. Analysis of gene expression changes induced by bexarotene in mammary gland revealed that 89 genes were dysregulated among the three transgenic mouse mammary models. From these, 9 genes were common to the three models studied.

**Conclusion:**

Analysis of the indicated core of transcripts and protein-protein interactions of this commonly modulated genes indicate two functional modules significantly affected by rexinoid bexarotene related to protein biosynthesis and bioenergetics signatures, in addition to the targeting of cancer-causing genes related with cell proliferation, differentiation and apoptosis.

## Background

The American Cancer Society estimates that 212,920 new cases of invasive breast cancer and 40,970 deaths were expected to occur in the United States in 2006 [1]. Approximately two-thirds of all breast cancers are ERα (+) at the time of diagnosis and expression of this receptor is determinant of a tumor phenotype that is associated with hormone-responsiveness. Patients with tumors that express ERα have a longer disease-free interval and overall survival than patients with tumors that lack ERα expression [2]. Despite the effectiveness of anti-estrogen selective ER modulators (tamoxifen and raloxifene) for ERα (+) breast cancer treatment, there is a clear need to develop agents for the prevention and treatment of ERα (-) breast cancer.

Genetically engineered mouse mammary cancer models are defined by a known genetic background and develop tumors after a predictable time course [3]. Importantly, mammary tumors arising in transgenic mice are generally ERα (-) providing a useful system for testing chemopreventive agents against hormonally non-responsive tumors.

Retinoids are biologically active derivatives of vitamin A that play essential roles in embryonic or adult cell behavior modulating cell proliferation, differentiation and apoptosis. Signal transduction is mediated by two classes of nuclear receptors retinoid-dependent transcriptional activators: the retinoic acid receptor (RARα, β, γ) and the retinoid × receptor (RXRα, β, γ). These ligand-depended transcription factors bind to response elements (RAREs or RXREs) in the promoter region of modulated genes [4]. The RXR protein can also dimerize with other nuclear hormone receptors such as vitamin D receptor, thyroid hormone receptors, PPAR (α, γ) and orphan receptors conferring rexinoids responsiveness to additional subset of target genes [5].

We previously analyzed the chemopreventive effectiveness of a highly selective RXR agonist, the rexinoid bexarotene (LGD1069) in three different transgenic mouse mammary models [6,7]. These studies showed a significant decrease in mammary tumorigenicity when MMTV-ErbB2, p53-null and C3(1)-SV40 tag mammary gland recipient virgin mice were treated with bexarotene (100 mg/kg dose). Although, bexarotene is more effective against c-erbB2 induced mammary tumors than against p53-null or SV40Tag mammary tumors; this data demonstrated that bexarotene is effective against the early stages of premalignant development independently of the genetic model assessed. More importantly, if specific gene expression signatures modulated by bexarotene across mammary cancer models could be identified, they might point to core transcriptional program/s on which attention should be focused.

In an effort to elucidate the molecular mechanism of action of chemopreventive rexinoid bexarotene and to identify potential biomarkers of significance, here we report a comparative transcriptome profiling of three mouse mammary cancer models by *Serial Analysis of Gene Expression *(SAGE). We focused our analysis on untreated mammary gland and on rexinoid bexarotene treated mammary gland at time periods prior to the histopathologic identification of premalignant progression. These studies identified a series of rexinoid-regulated genes and molecular pathways that may be critical for the cancer preventive activity of bexarotene.

## Methods

### Rexinoid LGD1069 and transgenic mouse mammary models

The RXR-selective retinoid used in this study bexarotene (LGD1069, Targretin) was obtained from Ligand Pharmaceutical, Inc (San Diego, CA).

Female MMTV-erbB2 mice [8], (obtained from The Jackson Lab., Bar Harbor ME) and C3(1)/SV40 T-antigen strain mice [9] (obtained from The National Cancer Institute, Frederick, MD) were housed in the institutional animal facilities. Balb/c p53-null mammary epithelium transplanted into the cleared mammary fat pads of three-week old mice p53 wt Balb/c mice [10] were initiated and maintained at BMC. Each group included age-matched untreated controls and bexarotene-treated mice. All mice were treated 6 days/week during 2 months starting at 8 weeks of age with bexarotene suspended in purified sesame oil (Croda, Inc., Mill Hall, PA). The retinoid was administered by gastric gavage using a 20-gauge gavage needle in a volume of 0.1-ml containing vehicle 100 mg/kg of bexarotene. Virgin animals were used to avoid confounding effects of hormonal surges during pregnancy. All animal research was conducted in AAALAC accredited facilities, following international guidelines and all research was approved by the corresponding Institutional bioethics committees.

### SAGE methodology

The six mouse SAGE libraries were generated following standard procedures as described previously [11]. Briefly total RNA was extracted from frozen samples using TRIzol (Invitrogen, Carlsbad, CA, USA). SAGE library construction was performed with the I-SAGE kit (Invitrogen, Carlsbad, CA, USA) according to the manufacturer's protocol and introducing only minor modifications. The anchoring enzyme was *Nla*III and the tagging enzyme used was *Bsm*FI. Concatemerized ditags were cloned into pZERO-1 and sequenced with an ABI 3700 DNA Analyzer (Applied Biosystems, Foster City, CA, USA). To decrease the chances potential artifacts due to sample heterogeneity, each control or bexarotene treatment SAGE library represents a pool of three mammary epithelial samples from three age-matched separate mice. For the studies on the p53-null mammary cancer model we used mammary epithelial enriched preparations as previously described [12], for the MMTV-erbB2 and C3(1)/SV40 T-antigen models we used total mammary gland preparations. SAGE libraries were generated at an approximate resolution of 60,000 SAGE tags per library.

### SAGE data processing and statistical analysis

SAGE tag extraction from sequencing files was performed by using the SAGE2000 software version 4.0 (a kind gift of Dr. Kenneth Kinzler, John Hopkins University, Baltimore, MD). SAGE data management, tag to gene matching, as well as additional gene annotations and links to publicly available resources such as Gene Ontology (GO), UniGene, and Entrez gene ID, were performed using a suite of web-based SAGE library tools developed by us. In our analyses we only considered tags with single tag-to gene reliable matches. To compare the control (vehicle) vs. bexarotene treatment SAGE libraries in each transgenic mice model, we utilized the Audic and Claverie's significance test [13]. Statistical analysis and scatter plot visualization of SAGE libraries were done with the Discovery Space 4 software (Genome Science Centre, BC Cancer Agency, Canada, Vancouver) .

### Bexarotene molecular signature determination

**Figure 1:**
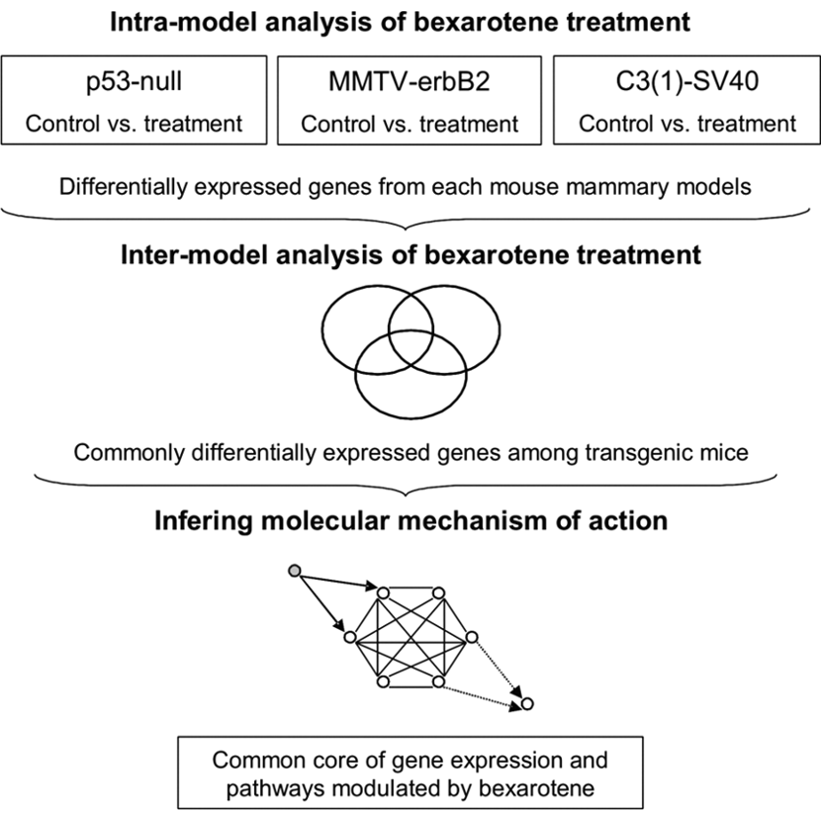
**Candidate genes and pathways modulated in normal mammary epithelium by rexinoid bexarotene in three different transgenic mice mammary cell models were identified through a three-stage process:A**. Identification of differentially expressed genes in mammary gland as a result of treatment with bexarotene comparing with vehicle control, in each of the mammary cancer models **B**. Inter-model comparison for the identification of overlapping gene expression profiles. **C**. Identification of associated functional modules and pathways affected by bexarotene treatment.

The main strategy of this analysis was to identify commonly deregulated genes by bexarotene treatment among the different mammary cancer models tested (Figure 1). Differentially expressed genes were compiled into one Excel spreadsheet pivot Table for comparison of overlapping data between p53-null, MMTV-erbB2 and C3(1)/SV40 T-antigen transgenic mouse mammary models. Any combination of two lists was compared for matching gene-identity. The number and identity of genes commonly affected in two models (*e.g*. MMTV-erbB2 vs. p53-null) was determined. We used the normal approximation to the binomial distribution as previously described [14] to calculate whether the number of matching genes derived from each pairwise comparison was of statistical significance (p < 0.05). To enable illustration of the commonly deregulated genes between mammary cancer models, we used the TIGR MultiExperiment Viewer (MeV 3.0) software. This tool was used for average clustering of SAGE based on the fold change of tag counts for each transcript comparing bexarotene treatment to control (vehicle) in each transgenic mice mammary model. For automated functional annotation and classification of genes of interest based on Gene Ontology (GO) terms, we used the *EASE *[15] available at the *Database for Annotation, Visualization and Integrated Discovery *(*DAVID*) [16]. All of the raw SAGE data reported as additional files in this article are publicly available and also can be viewed at .

In order to identify the molecular pathways that are mainly affected by the rexinoid bexarotene, we look for protein/gene interaction networks in the common core of modulated genes. The protein-protein interaction network associating genes of the three transgenic mouse mammary models was generated using the database STRING ('Search Tool for the Retrieval of Interacting Genes/Proteins') [17]. The database STRING aims to collect, predict and unify most type of protein-protein associations, including direct and indirect associations. STRING runs a set of prediction algorithms, and transfers known interactions from model organisms to other species based on predicted orthology of the respective proteins [18]. In order to identify each gene in the database, we used both mouse gene name and Entrez gene ID in the 'protein-mode' application. The analysis input options were 'co-occurrence', 'co-expression', 'experiments', 'databases', and 'text mining' data at high confidence level of predicted human orthology groups. Pathways are discriminated by different colors based on up-modulated (red node) or down-modulated (green node) transcripts in order to indicate protein-protein networks modulated by bexarotene across mammary cancer models.

## Results and discussion

RXR-selective rexinoids inhibit the proliferation of normal, pre-malignant and malignant breast cells suppressing mammary tumor development in MMTV-erbB2, p53-Null, and C3(1)/SV40 T-antigen transgenic mice models [[6,7] and Medina et al., unpublished]. The chemopreventive effects of bexarotene are likely due to transcriptional modulation of genes related to repression of cell proliferation and stimulation of apoptosis and cell differentiation [19].

**Figure 2:**
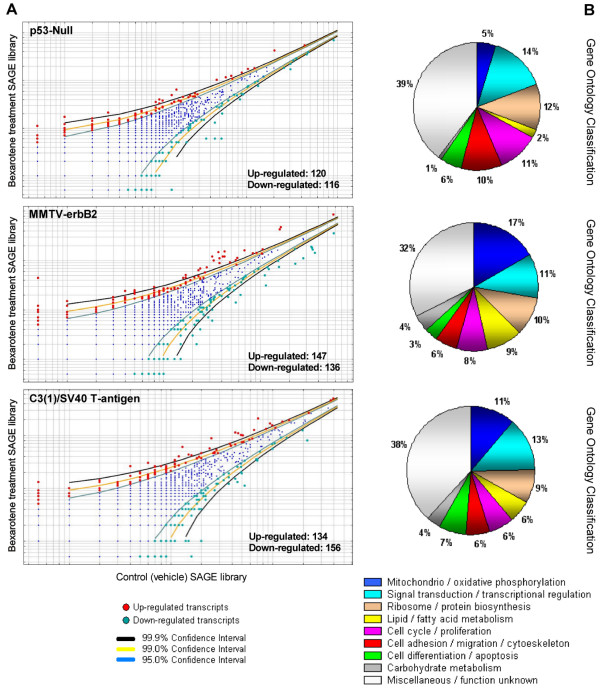
**Deregulated transcripts in mammary gland by systemic treatment with bexarotene in the three transgenic mice mammary cancer models.A**. Scatter-plot representation of differentially expressed genes between bexarotene treated mice and vehicle control SAGE libraries (p < 0.05). **B**. Gene ontology (GO) classification of bexarotene induced differentially expressed transcripts on mammary gland from the different transgenic models. Relative representation of the deregulated transcripts with specific GO term annotations related to biological processes or molecular function.

In order to identify rexinoid-regulated biomarkers, we generated six mouse SAGE libraries corresponding to mammary gland samples from control and bexarotene treatment from three transgenic mouse mammary cancer models: p53-Null [10], MMTV-erbB2 [8] and C3(1)/SV40 T-antigen [9]. This resulted in the sequencing of 360,000 tags (60,000 tags per library), thus monitoring the behavior of more than 20,000 transcript tags. Our statistical analyses revealed 236 transcripts differentially regulated by bexarotene treatment in mammary epithelium from p53-null background, 283 transcripts in mammary gland from the MMTV-erbB2 model, and 290 transcripts in the C3(1)/SV40 T-antigen transgenic mice mammary model (Figure 2A; see Additional file 1). Table 1 shown the most highly bexarotene deregulated transcripts from each transgenic mice mammary cancer model (Fold change ≥ 7; p < 0.01).

**Table 1:** Most highly deregulated transcripts in mammary gland induced by bexarotene treatment on each transgenic mice mammary cancer model (Fold change ≥ 7; p < 0.01).

**Tag**	**Gene**	**Description**	**Entrez Gene**	Fold Change*
***p53-null***				
GTTTGCTGTA	*Serpinb6a*	*Serine (or cysteine) peptidase inhibitor*	20719	17.0
AGTCTCGAGG	*Slc1a5*	*Solute carrier family 1*	20514	12.0
GGTTTGGGGG	*Jup*	*Junction plakoglobin*	16480	11.0
TGCGTGCTGG	*Timp2*	*Tissue inhibitor of metalloproteinase 2*	21858	11.0
TTGAAATTAC	*BC061494*	*CDNA sequence*	381832	11.0
GATTTCTTTG	*Gpc3*	*Glypican 3*	14734	10.0
TAACCAAAAA	*Itgb4*	*Integrin beta 4*	192897	10.0
CCCAGTCCCT	*Ltbp4*	*Latent transforming growth factor bindin. prot. 4*	108075	8.0

GACTCTATAT	*Csn2*	*Casein beta*	12991	-15.0
CAATAAAACA	*Sar1b*	*SAR1 gene homolog B (S. Cerevisiae)*	66397	-11.0
GCAGCGATTC	*Nme2*	*Expressed in non-metastatic cells 2*	18103	-10.0
TGTTCTATGG	*Laptm5*	*Lysosomal-associated protein transmembrane 5*	16792	-9.0
GTGTTTTGCT	*AI451557*	*Expressed sequence*	102084	-9.0
CTAGGTGGTG	*Glycam1*	*Glycosylation dependent cell adhesion molecule 1*	14663	-8.8
TAAAGTCAAT	*Muc15*	*Mucin15*	269328	-8.0
TCAGAGTGAG	*Igh-6*	*Immunoglobulin heavy chain 6*	16019	-7.5

***MMTV-erbB2***				
AGACCCTGTC	*Pnpla3*	*Patatin-like phospholipase domain containing 3*	116939	44.0
TATGAGATAG	*Timm9*	*Translocase of inner mitochondrial membrane 9*	30056	15.0
AGCCCTCGGA	*Acads*	*Acyl-Coenzyme A dehydrogenase*	11409	12.0
ACCGGGCTGG	*Elovl6*	*ELOVL family member 6*	170439	12.0
TGACAGAAGA	*Tnnc2*	*Troponin C2*	21925	10.0
TCTCTCAGTC	*Anxa5*	*Annexin A5*	11747	9.0
CACAGAACCA	*0610031J06*	*RIKEN cDNA 0610031J06 gene*	56700	7.0
CCTGCAGCAG	*2900073H19*	*RIKEN cDNA 2900073H19 gene*	68205	7.0

GCCACTTAAG	*Cd79a*	*CD79A antigen (immunoglubulin-associated alpha)*	12518	-15.0
AGCCATCATA	*2610042O14*	*RIKEN cDNA 2610042O14 gene*	66460	-13.0
AGCGAAATAA	*Gmfg*	*Glia maturation factor gamma*	63986	-11.0
CTGCAGCCTA	*Stx5a*	*Syntaxin 5A*	56389	-11.0
TTACAAGCCT	*Cks1b*	*CDC28 protein kinase 1b*	54124	-10.0
GTGGACTCAA	*Ifitm1*	*Interferon induced transmembrane protein 1*	68713	-10.0
CATAGTTTAA	*Nol7*	*Nucleolar protein 7*	70078	-10.0
AAGTTCTTCA	*Csn1s2a*	*Casein alpha s2-like A*	12993	-9.0

***C3(1) SV40 T-antigen ***				
AGCAGTGCTT	*Ccdc3*	*Coiled-coil domain containing 3*	74186	13.0
CAGTTTGTAA	*Pdha1*	*Pyruvate dehydrogenase E1 alpha 1*	18597	10.0
AATGTGTATG	*Abca8a*	*ATP-binding cassette sub-family A (ABC1)*	217258	9.0
ATTCCCTGTT	*Krtap8-1*	*Keratin associated protein 8-1*	16703	8.0
CCGAAAAAAA	*Pink1*	*PTEN induced putative kinase 1*	68943	7.0
ACTCTAAAAA	*Tmem55b*	*Transmembrane protein 55b*	219024	7.0
CTGTAGTGTC	*Ltf*	*Lactotransferrin*	17002	7.0
CTGTCCAAGG	*Bhlhb2*	*Basic helix-loop-helix domain containing class B2*	20893	7.0

GAAAATAAAA	*Fndc3a*	*Fibronectin type III domaing containing 3a*	319448	-20.0
TAAATTAAGA	*Hexb*	*Hexosaminidase B*	15212	-16.0
TTAGAAGTGA	*Sav1*	*Salvador homolog 1 (Drosophila)*	64010	-15.0
GGGGGTGAGG	*Hisppd1*	*Histidene acid phosphatase domain containing 1*	227399	-15.0
TAACAAAGGA	*Ahcyl1*	*S-adenosylhomocysteine hydrolase-like 1*	229709	-14.0
GATTAAAACA	*4931406I20*	*RIKEN cDNA 4931406I20 gene*	66743	-11.0
TTAACACTGT	*Rab35*	*RAB35, member RAS oncogene family*	77407	-10.0
CAGATTAAAA	*Gbp6*	*Guanylate binding protein 6*	229900	-9.0

*Up-regulated transcripts in bexarotene treatment are represented by positive fold changes and down-regulated transcripts are represented by negative fold changes.

**Figure 3:**
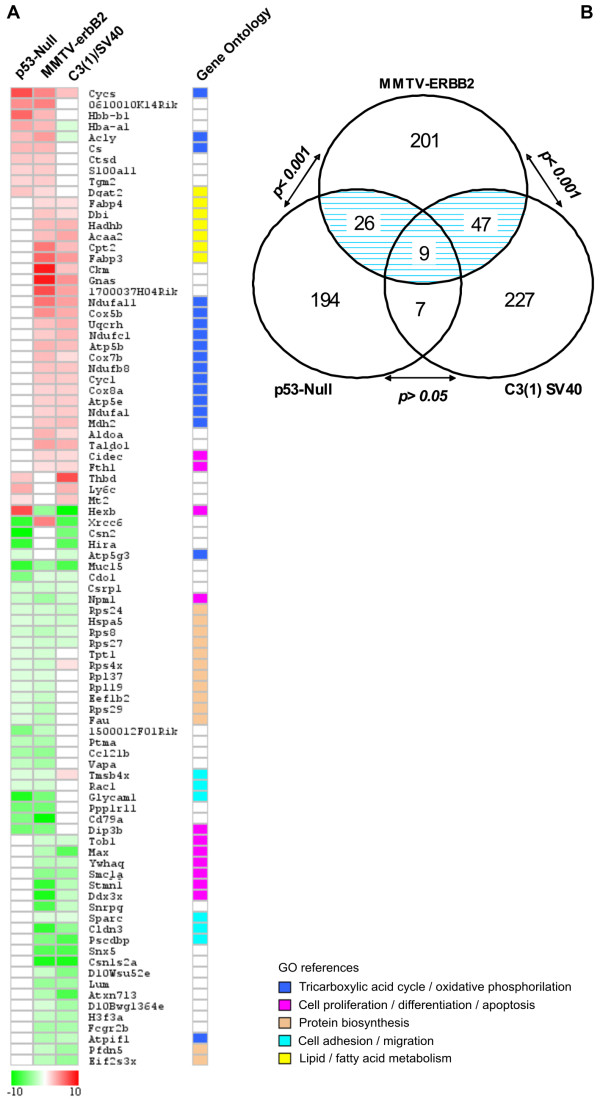
**Co-occurring differentially expressed genes among transgenic mouse mammary models.** Eighty-nine genes were identified as modulated in more than one transgenic mice model. **A**. Heat map of the 89 deregulated transcripts. Color scale at the bottom depicts the approximate fold change in expression for each transcript and library relative to control mammary gland. Negative fold change (transcripts with decreased expression in bexarotene treated animals) is represented in green, and positive fold change (transcripts with overexpression in bexarotene treated mice) is represented in red. **B**. Statistical comparison between MMTV-erbB2 vs. p53-null and MMTV-erbB2 vs. C3(1)/SV40 T-antigen transgenic mice models showing a highly significant number of overlapping genes (p < 0.001). The number of overlapping genes between p53-null and C3(1) SV40 models it is not statistical significant (p > 0.05).

In order to identify co-occurring differentially expressed genes among the three transgenic mice analyses, we performed an inter-model comparison between the above-described SAGE datasets (Figure 1). Among the three mice mammary models, a total of 711 transcripts were identified as deregulated by the rexinoid bexarotene treatment. Eighty-nine genes were identified in more than one mammary cancer model (Figure 3A; see Additional file 2). Interestingly, nine of these 89 genes were deregulated by bexarotene in mammary gland tissue from all three transgenic models: *Muc15 *(*Mucin 15*), *Cdo1 *(*Cystein dioxygenase 1*), *Rps8 *(*Ribosomal protein S28*), *Rps27 *(*Ribosomal protein S27*), *Rps24 *(*Ribosomal protein S24*), *Hspa5 *(*Heat shock 70 kD protein 5*), *Csrp1 *(*Cysteine and glycine-rich protein 1*), *Npm1 *(*Nucleophosmin 1*), and *Cycs *(*Cytochrome c somatic*). Gene Ontology annotation of the 89 deregulated genes that were common in any two models showed that approximately 18% of the transcripts are involved in *tricarboxylic acid cycle/oxidative phosphorylation*, 14% are related to *signal transduction/transcriptional regulation*, 14% are related to *protein metabolism *and 12% are related to *cell proliferation/differentiation and apoptosis*.

A probabilistic analysis showed that 56 genes were co-deregulated in MMTV-erbB2 and C3(1)/SV40 T-antigen mice models, representing a non-random significant number of overlapping genes based on normal approximation to the binomial distribution (p < 0.001) (Figure 3B). Thirty-five genes were identified as co-deregulated in MMTV-erbB2 and p53-null mice models (p < 0.001). The set of 16 genes overlapping between p53-null and C3(1)/SV40 T-antigen were not statistical significant, i.e. the overlapping could be simply by chance (p > 0.05) (Figure 3B). In other words, it appears that a better correlation was observed between MMTV-ErbB2 with the other two models, than between p53-null and C3(1)-SV40 tag transgenic mouse mammary gland models. These data suggest that mammary tumors derived from different primary oncogenic pathways could respond differently to the same chemoprevention agent. In addition, these results indicates that transcripts modulated by bexarotene in the MMTV-ErbB2 mammary gland share almost all the common features among the transgenic mouse models analyzed. As mentioned above, we have previously shown that bexarotene suppresses mammary tumor development in the MMTV-ErbB2, p53-null and C3(1)-SV40 tag transgenic mouse mammary gland models [6,7]. Interestingly, the specific response of these three transgenic mouse mammary models to bexarotene treatment varies with the genetic background assessed. For instance, the bexarotene treatment is much more effective against MMTV-ErbB2 induced mammary tumors than against C3(1)-SV40 or p53-null mammary tumors [Medina et al., unpublished]. In the MMTV-ErbB2 mammary gland, bexarotene reduced tumor incidence by 75% and lengthened median tumor latency from 234 days to over 420 days [7]. However, in the p53-null and C3(1)-SV40 mammary gland where p53 or p53/Rb activities are affected respectively, bexarotene treatment showed modest chemoprevention activity. Both these molecules exert primary functions downstream of the CDKs, loci of targets activity. In this sense, human breast cancer is a complex disease caused by dysregulation of many different oncogenes, tumor suppressor genes and growth factor pathways. The MMTV-ErbB2, p53-null and C3(1)-SV40 tag mouse mammary gland cancer models are valuable tools for the elucidation of the mechanisms of mammary tumorigenesis [3]. However, it is important to recognize that no one model represents the heterogeneity of human breast cancer.

**Figure 4:**
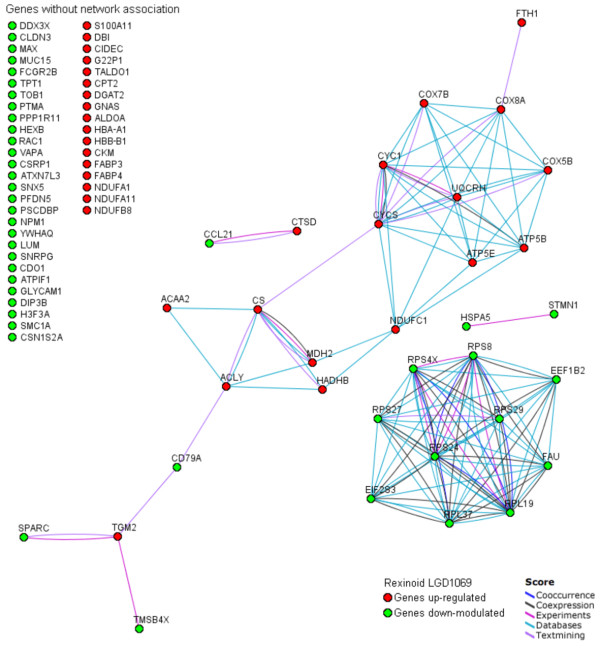
**Graph of interactions among the common core of genes modulated by rexinoid bexarotene in the different mammary mice genetic models generated using database STRING.** Genes without known interactions with other genes are listed in the left of the figure. In the network: links between proteins means the various interactions data supporting the network, colored by evidence type.

We present in Figure 4 a protein-protein interaction network associating the common core of non-random bexarotene modulated genes across transgenic mouse mammary models. The graph was generated employing the STRING on-line resource based on high confidence data related with 'co-expression/co-ocurrence', 'experimental/biochemical data' and 'association in curated database/text mining' [17]. STRING is a comprehensive tool integrating protein association information with the capability to transfer known interactions from model organisms to other species (*e.g*.: from mouse to human orthology genes/proteins) based on predicted orthology of the respective proteins. The generated graph (Figure 4) indicates strong interactions among a set of 33 proteins transcriptionally modulated by bexarotene. Furthermore, the network architecture suggests the existence of two functional modules in this figure, involving the down-modulation of genes related with *protein biosynthesis *pathway, and up-modulation of genes related with *tricarboxylic acid cycle/oxidative phosphorilation *pathways.

### Protein biosynthesis signature

A common observation in cancer gene expression profiling is the systematic up-regulation of ribosomal genes among the most abundant transcripts in human and mouse mammary carcinomas compared with normal tissues [20-24]. The up-regulation of ribosomal genes was significantly correlated with variation in the cell doubling time *in vitro*, supporting the notion that these genes are up-regulated in relation to the increase of cell proliferation rate or growth rate during malignant transformation. Interestingly and in an opposite manner, bexarotene treatment cause in 'normal' mammary gland the down-regulated expression of more than 10 genes related to protein biosynthesis including numerous ribosomal proteins (*Rpl19*, *Rpl37*, *Rps4x*, *Rps8*, *Rps24*, *Rps27*, *Rps29*), *Eef1b2 *(*Eukaryotic translation elongation factor 1 beta 2*), *Eif2s3x *(*Eukaryotic translation initiation factor 2*), *Fau *(*Finkel-Biskis-Reilly murine sarcoma virus*) and *Tpt1 *(*tumor protein, translationally-controlled 1*). The inhibition of mRNA synthesis for genes encoding ribosomal proteins has been suggested as a mechanism that could reprogram the cancer cell to recover some of its normal functions in a tumor reversion process [25].

*Tpt1 *(also known as *Tctp*) encodes a GDP dissociation inhibitor protein of the translation elongation factor eEF1A [26]. The human *TPT1 *gene is overexpressed in cancerous cell lines compared with cell lines derived from normal tissues. Tuynder et al. (2002) demonstrated that the expression levels of TPT1 were strongly down-regulated at the mRNA and protein levels during tumor reversion/suppression. MCF7 and T47D cell lines transfected with Tpt1 siRNA showed a more organized ductal-like structures similar to those generated by down-regulation of β1 integrin [25]. Here we observed that bexarotene significantly dowregulated expression of *Tpt1 *in mammary epithelium.

### Bioenergetics signatures

More than 50 years ago, Warburg proposed that malignant phenotype might be caused by a decrease in mitochondrial energy metabolism paralleled by increased glycolytic flux [27]. Increasing evidence is in line with this hypothesis suggesting a close link between metabolic and genetic changes observed during malignant growth [28,29]. Recently it has been demonstrated that impaired bioenergetic function of mitochondria is a hallmark of carcinogenesis in breast, gastric, lung and oesophageal cancer [30,31]. Moreover, Schulz et al. (2006) showed that induction of mitochondrial oxidative metabolism efficiently suppresses malignant growth *in vitro *and *in vivo*. Interestingly, we identified a systematic up-regulation of transcripts related to oxidative phosphorylation induced by bexarotene treatment in mammary gland (Figure 4). The transcripts commonly up-regulated by bexarotene treatment in at least two of the models were *Atp5b *(*ATP synthase F1 complex beta subunit*), *Atp5e *(*ATP synthase F1 complex epsilon subunit*), *Cyc1 *(*Cytochrome c-1*), *Cycs *(*Cytochrome c somatic*), *Cox5b *(*Cytochrome c oxidase, subunit Vb*), *Cox7b *(*Cytochrome c oxidase subunit VIIb*), *Cox8a *(*Cytochrome c oxidase, subunit VIIIa*), *Ndufa1 *(*NADH dehydrogenase 1 alpha subcomplex*), *Ndufc1 *(*NADH dehydrogenase 1*), *Ndufb8 *(*NADH dehydrogenase 1 beta subcomplex 8*), *Ndufa11 *(*NADH dehydrogenase 1 alpha subcomplex 11*) and *Uqcrh *(*Ubiquinol-cytochrome c reductase hinge protein*) (see Additional files 1 and 2). Consistent with a significant increase of oxidative phosphorylation enzymes, we observed that *Atpif1 *gene (*ATPase inhibitory factor 1*) was significantly down-regulated by bexarotene treatment in the MMTV-erbB2 and C3(1)/SV40 T-antigen transgenic mice models. In this sense, Isidoro et al. (2005) showed that down-regulation of ATPase β-F1 per se allowed the identification of a subgroup of breast cancer patients with significant worse prognosis. Finally, is important to note that mitochondrial oxidative phosphorylation is required for efficient execution of apoptosis. Cells which are unable to carry on oxidative phosphorylation have a resistant apoptotic phenotype [32]. Overall, these findings suggest the oxidative phosphorylation induction (prevention impaired bioenergetic function) as a novel mechanism of bexarotene's chemopreventive effects.

### Fatty acid metabolic signature

Lipid metabolism and the intracellular transport of bioactive species is a critical component in the process by which these molecules continuously stimulate proliferation through interactions with nuclear receptors. Bexarotene treatment of MMTV-ErbB2 and C3(1)/SV40 transgenic mammary gland up-regulated various genes related with lipid/fatty acid metabolism (Figure 4) such as: *Fabp3 *(*Fatty acid binding protein 3*), *Fabp4 *(*Fatty acid binding protein 4*), *Dgat2 *(*Diacylglycerol O-acyltransferase 2*), *Dbi *(*Diazepam binding inhibitor*), *Hadhb *(*Hydroxyacyl-Coenzyme A dehydrogenase*), *Acca2 *(*Acetyl-Coenzyme A acyltransferase 2*), and Cpt2 (Carnitine palmitoytransferase 2). Interestingly, a family of cytoplasmic proteins known as FABPs mediates transport and utilization of lipids, and different FABP types have been implicated in control of cell proliferation and cancer progression. Recently, FABP1 and FABP2 were shown to be up-regulated in breast cancer cell lines while FABP3 and FABP4 were down-regulated in breast cancer cells [33]. Moreover, FABP4 is a marker protein for differentiated mammary gland that is expressed only in normal lactating cells and not in tumor mammary cells. Transfection of cDNA clone of FABP4 into MCF7 cells results in growth inhibition and lower tumorgenicity in nude mice [34].

### Cell proliferation and apoptosis signatures

Rexinoid bexarotene down-regulated several genes related with cell cycle/proliferation in mammary gland from the different transgenic mice models (Figure 2A; see Additional files 1 and 2).

Among this functional group we find *Npm1 *(also known as Nucleophosmin/B23) protein that belongs to a nuclear chaperone family of phosphoproteins that take part in various cellular processes such as cell proliferation and transformation [35]. Human *NPM1 *is overexpressed in various tumors types, and it has been proposed as a marker for gastric, colon, ovarian and prostate carcinomas [35]. *NPM1 *overexpression promotes cell survival in several cell types through the inhibition of distinct pro-apoptotic pathways [36]. We detected a systematic down-regulation of *Npm1 *gene in mammary gland from Bexarotene treated mice on the three models studied (average fold change = -2.6; p < 0.01). Interestingly, proteomic analyses identified NPM1 as a protein associated with acquired estrogen-independence in human breast cancer cells [37]. Moreover, down-regulation of *NPM1 *mRNA delay cell-cycle progression and the entry into mitosis [38], whereas *NPM1 *overexpression decreases the sensitivity of human leukaemia cells to retinoic-acid-induced differentiation and apoptosis [39,40].

Another gene in this category includes *Stmn1 *which encodes an 18 kDa cytosolic phosphoprotein (also known as Stathmin 1 or Oncoprotein 18) that is regulated during cell cycle by transcriptional and posttranscriptional mechanisms. *STMN1 *overexpression has been demonstrated at mRNA and protein levels in a significant proportion of human breast carcinomas (about 30%) [41]. Moreover, *STMN1 *overexpression was correlated with the loss of ERα and with histological grade III breast carcinomas. STMN1 has been suggested as a key regulator of the cell division through its influence on microtubule dynamics. We identified a statistical significant decrease of *Stmn1 *expression (average fold change = -5.4; p < 0.05) caused by bexarotene treatment in mammary gland from MMTV-erbB2 and C3(1)/SV40 T-antigen mice. Interestingly, we previously demonstrated that mouse *Stmn1 *and human homologue *STMN1 *genes are overexpressed in invasive breast carcinomas by northern and real time RT-PCR analyses [24].

Numerous studies have shown that down-regulation of p27^Kip1^, an *inhibitor of cyclin-dependent kinase*, is associated with poor prognosis in many cancers such as: breast, colorectal, prostate, and lung carcinomas. We previously detected the overexpression of *CDC28 protein kinase regulatory subunit 1B (Cks1b*) in human and mouse mammary tumors [24]. Interestingly, rexinoid bexarotene strongly down-regulated *Cks1b *expression in the MMTV-erbB2 model (Fold change = -10.0; p = 0.006) (see Additional file 1). Human *CKS1B *functions as an important adaptor of SCF Skp2 ubiquitin ligase and facilitates SCF Skp2 targeting of the cell proliferation inhibitor p27 (Kip1) for ubiquitination and subsequent degradation [42]. It was also suggested that CKS1B may be involved in p21 degradation in a similar fashion [43]. Overexpression of *CKS1B *has been observed associated to poorly differentiated tumors (histological grade III) and with the loss of ER/PR status [Slotky et al., 2005]. In addition, *CKS1B *overexpression was strongly and independently associated with poor overall survival in human breast cancer [44].

On the other hand, bexarotene treatment up-modulated two apoptotis related genes (*Cidec and Cycs*) in 'normal' mouse mammary gland from two of the models (*Cidec*) and in all three models (*Cycs*) (Figure 3). *Cidec *(also known as *Fsp27*) encode a novel family member of the cell-death-inducing DFF45-like-effectors (CIDEs) [45]. Although, its well known that DFF45 is a subunit of the DNA fragmentation factor that is cleaved by caspase-3 during apoptosis [46]. The molecular mechanism by which Cidec induces apoptosis remains to be elucidated.

### Cell adhesion and invasion signatures

During their metastatic conversion, epithelial cells acquire the ability to invade the surrounding tissues and later disseminate to secondary organs mostly via lymphatic vessels. Epithelial cell adhesions, including intercellular (junctional) and cell-extracellular matrix adhesions, are critical to the maintenance of structural integrity, polarity, and cell-cell communication. We detected a significant decrease in *Cldn3 *(*Claudin 3*) (Average fold change = -6), *Glycam1 *(*Glycosylation dependent cell adhesion molecule 1*) (Average fold change = -7), *Pscdbp *(*Pleckstrin homogy Sec7 binding protein*) (Average fold change = -6) gene expression modulated by bexarotene treatment among transgenic mice models. The *Claudin *genes (*Cldn*) encode a family of proteins important in epithelial cell tight junction, which are critical to the maintenance of cell polarity and permeability [47,48]. Most Claudin genes appear with decreased expression in cancer however *CLDN3 *and *CLDN4 *genes have been found frequently up-regulated in ovarian, breast, prostate and pancreatic cancers [49-52]. Recently, has been suggested that Claudins may be envolved in survival and invasion of cancer cells [48]. We detected down-regulation of *Cldn3 *gene in mammary gland from bexarotene treated mice in the MMTV-erbB2 and C3(1)/SV40 models. The role of *Gycam1 *and *Pscdbp *genes in breast cancer progression remains unknowns.

## Conclusion

The present study showed that the rexinoid bexarotene (LGD1069) exerts its chemopreventive activity by affecting multiple cellular pathways, not only targeting cancer-causing genes related with cell proliferation, differentiation and apoptosis, but also by modulating protein biosynthesis and mitochondrial bioenergetics. Further analysis and validation of the identified genes will be required to determine the prognostic value as biomarkers of bexarotene treatment response, and to determine whether some of them and their protein products may constitute novel candidates for additional targeted therapeutic interventions.

We have recently completed a Phase II biomarker modulation trial in which women at high risk of breast cancer were treated with placebo or bexarotene. Using breast tissue from these high risk women, we are now studying whether these newly identified rexinoid-regulated biomarkers are also being modulated in human breast tissue. Results from these human studies will reveal whether these new biomarkers will be useful for predicting a cancer preventive response from rexinoids or as targets for future therapies.

## Abbreviations

SAGE: Serial Analysis of Gene Expression; ER: Estrogen Receptor; RAR: Retinoic Acid Receptor; RXR: Retinoid × Receptor; GO: Gene Ontology database; DAVID: Database for Annotation, Visualization and Integratid Discovery; STRING: Search Tool for the Retrieval of Interacting Genes/Proteins.

## Competing interests

The authors declare that they have no competing financial interests. As indicated author Reid Bissonnette works for Ligand Pharmaceuticals.

## Authors' contributions

MCA conducted the analysis of the data; wrote the article; YH and CCL conducted SAGE studies; SG in charge of bioinformatics resources; FSK, YZ, JH in charge of animal experiments; RPB provided the compound LGD1069 and contributed to the writing of the study, DM contributed to the writing of the manuscript and directed the p53 null studies, PHB and CMA directed the studies and contributed to the writing of the article.

## Pre-publication history

The pre-publication history for this paper can be accessed here:



## Supplementary Material

Additional file 1 Differentially expressed genes in mammary gland as the result of systemic bexarotene treatment versus control. The data provided represent the statistical analysis of SAGE libraries from p53-Null, MMTV-erbB2 and C3(1)/SV40 transgenic mice mammary cancer models (p < 0.05). Click here for file

Additional file 2 Co-occurring mammary gland deregulated transcripts as the result of systemic bexarotene treatment among transgenics mice mammary cancer models. The data provided represent the inter-model comparison for the identification of overlapping gene expression profiles among transgenics mice mammary cancer models (p < 0.05). Click here for file
